# From Moses to Moses

**DOI:** 10.5041/RMMJ.10013

**Published:** 2010-10-31

**Authors:** Menachem Kellner

**Affiliations:** Department of Jewish History, University of Haifa and Merkaz Shalem, Jerusalem, Israel

Let us begin with a thought experiment. What would Judaism look like today had Maimonides not lived?

Had he not created the first systematic and comprehensive code of Jewish law (*Mishneh Torah*) would his successors in that project, R. Jacob ben Asher, author of the *Arba’ah Turim*, and R. Joseph Karo, author of the *Shulhan Arukh*, have had the vision and courage to embark on what would have been, if not for Maimonides, a revolutionary innovation? The *Mishneh Torah* is revolutionary in three ways: it was comprehensive, covering every aspect of Jewish law, including vast areas of Jewish practice which, in Maimonides’ day, were simply inapplicable; it was systematic, almost geometrical in its approach (I like to tell my students that the *Mishneh Torah* involves the application of Greek modes of thought – systematic, axiomatic – to Jewish content – halakhah); and it was an apodictic code, presenting the law in absolute terms, not Maimonides’ opinion about what the law should be. It is in every sense revolutionary, and without that revolutionary model it is unlikely that subsequent codifiers would have had the courage (and precedent) to do what they did.

Had Maimonides not placed Judaism on a firm dogmatic footing (with his “Thirteen Principles”),^1^,^2^ would it be possible to speak of Jewish *orthodoxy* (orthos + doxos = straight beliefs) in any technical sense of the term? Maimonides threw the massive weight of his rabbinic authority behind the claims 1) that Judaism had a category of commandments addressed to the intellect (in his *Duties of the Heart*, Bahya ibn Pakudah had made the claim earlier, but Maimonides formulated it more forcefully, more absolutely, and with much greater authority) and 2) that some of these commandments had the status of dogmas, in the strictest sense of the term. This second claim was absolutely unprecedented in Judaism and changed the face of the religion for ever. One example should suffice to prove the point: without Maimonides’ dogmas (*ikkarim*) David Berger’s jihad against Habad would not have a leg to stand on.^3^

Had not Maimonides thrown the massive weight of his considerable authority behind the project of integrating science and Judaism (in his *Guide of the Perplexed*) how much room would the Jewish world have made for rationally oriented Jews in the Middle Ages and today? The Jewish world in which we live is dramatically infused with a wide variety of kabbalistically inspired movements: Hasidism, almost all of Mizrahi and Lithuanian haredism, Greater Land of Israel doctrines, New Age spiritualities, etc.; also, the use of amulets, prayers at graves, (usually expensive) visits to wonder-working “rabbis”, the whole notion of rabbinic authority in non-halakhic spheres, etc. – without Maimonides’ authority it would be next to impossible to carve out a normative Jewish niche for those convinced that God gave humans brains to use in an independent and rational fashion. Had not Maimonides presented the Jewish world with an alternative to Kabbalah, would all Jews today embrace various offshoots of Kabbalistic Judaism?^4^

Alternatively, if Moshe Idel is correct, and Kabbalah “went public” in *response* to Maimonides,^5^ would the Jewish world be much less mystically oriented than it is today? According to Idel, Jews always engaged in mysticism, but quietly, unobtrusively, in secret. It was the challenge of Maimonides’ austere, rationalist Judaism (“It’s Greek to me!”, many of his contemporaries must have thought – and not been far wrong) which forced Kabbalah out of the subterranean channels in which it had hitherto flowed. If Idel’s analysis is correct, by forcing the Kabbalists to do battle with his philosophical Judaism, Maimonides ironically brought about its defeat – as no one who looks around the Jewish world today can possibly deny (more’s the pity!).

Further, and perhaps also ironically, Maimonides sought to lower messianic fervor by treating messianism in the most naturalistic way possible, as a process which takes place in *this* world, without overt divine intervention, and with no violations of natural law (yes, messianic lambs can dwell with messianic wolves, so long as you have a continuing supply of new lambs).^6^ It is this approach to the messiah which makes religious Zionism of the Kookian variety possible – for good or for ill, depending on your perspective. It takes a Maimonidean understanding of messianism to see draining swamps, building a secular state, establishing an army, etc., as stages in the *athalta de-ge’ulah* (beginning of redemption).^7^

Finally, had Maimonides not enunciated a universalist vision of Judaism would almost all Jews today be even more particularist than they are?^8^ Most Israeli secular Jews, and almost all Israeli orthodox Jews, as well as many secular Jews in the Diaspora and almost all orthodox Jews there, are convinced that there is something inherent, intrinsic, metaphysical, mystical (choose your favorite term) that distinguishes Jews from Gentiles; on this view, as my friend Professor Daniel J. Lasker likes to say, the difference between Jew and Gentile resides in their hardware and not only in the different software they “run”.^9^ This hard-wired version of particularism often leads to consequences I would rather not go into here. Maimonides, along with the prophet Isaiah and with God, is one of Judaism’s most out-spoken universalists: all human beings are truly created in the image of God, period.

There are, of course, no answers to the questions posed here – this is only a thought experiment, after all. But even as such it should be enough to make clear how little exaggeration there is in the famous saying, “From Moses [son of Amram] to Moses [son of Maimon] – there arose none like Moses!” With the exception, perhaps, of R. Judah the Prince, editor of the Mishnah, I can think of no single *individual* who lived between the two Moseses whose absence would be felt so dramatically had he not lived.

Our thought experiment shows that Maimonides is best understood as a revolutionary, but a revolutionary who in his own eyes was deeply conservative, seeking to save true Judaism, as he understood it, from generations of Jews and their rabbis who did not understand it. He had to tread, therefore, very carefully. In this, he was convinced that he followed the first Moses: Maimonides himself explains in the Introduction to the *Guide of the Perplexed* that the Torah has an esoteric level which it would be inappropriate to teach publicly. Like the first Moses, therefore, were he to write anything about Torah, Maimonides would have to write esoterically, i.e. to address dramatically different audiences simultaneously. As I often tell my students, Maimonides has to write both for my haredi friends and for me simultaneously, and write in such a fashion that we all believe that ours is the correct interpretation. What, then, is the secret that Maimonides hides?

Leo Strauss thought that it was that Maimonides was fundamentally an orthodox Aristotelian and thus only pretended to be what today would be called an orthodox Jew (Strauss was notoriously cagey about expressing his personal views. I found Leora Batnitzky^10^ very helpful in understanding Strauss).^10^,^11^ It is this approach which leads Shlomo Pines (in his introduction to his translation of the *Guide of the Perplexed*)^12^ to call Maimonides’ halakhic enterprise only an “avocation”. I do not believe that any close student of the *Mishneh Torah* – not to mention the Commentary on the Mishnah, the Book of Commandments, and the responsa – could be anything but amazed by this insouciant dismissal of that field to which Maimonides devoted most of his energy and most of his intellect throughout his life. Maimonides also devoted astonishing amounts of energy to the mundane affairs of the Egyptian Jewish community of his day.^13^–^15^ Is this the behavior of a man who believed that if immortality of any sort is possible, it depends upon the development of one’s intellect? Every moment spent away from philosophical reflection is a moment wasted, never to be regained. Let it also be noted that after Maimonides finished writing the *Guide of the Perplexed* in about 1191, he wrote no more philosophic or scientific texts, whereas he never ceased occupying himself with his “avocation”.

So, what is the secret that Maimonides hides? He himself tells us:^16^–^18^ the rabbis of the Talmud used the expression *ma’aseh bereshit* (“account of creation” in Genesis) for what the Greeks called physics and used the expression *ma’aseh merkavah* (“account of Ezekeiel’s chariot vision”) for what the Greeks called metaphysics. So why is this important? The consequences of these equations are momentous. Maimonides imports what we today would call science into the heart of Torah.^19^ This is allied to his universalism (Jews are distinguished from non-Jews *only* by behavior and belief (knowledge)) and to his conception of the commandments of the Torah as tools (which could in principle have been different), whose importance lies in the end they serve, and not in themselves. That being the case, true reward and punishment are not connected to behavior, no matter how saintly or how vile.

Maimonides hid these secrets from his fellow Jews, not out of fear of reprisal (protected as he was by his good friend, al-Qadi al-Fadl, Saladin’s vizier, he had no reason to fear them), but out of *noblesse oblige*. Exposing simple Jews (and their philosophically no less simple rabbis) to these truths could only lead to perplexity (in the best of circumstances) or to falling away from observance (in the worst of circumstances), neither of which Maimonides had any interest in promoting.

One God wrote two books, as it were: Torah and Cosmos. The truly devout Jew realizes that he or she must study both books, or only have access to half of God’s oeuvre. This is a secret which very few Jews – then or now – are comfortable accepting.

This vision of Maimonides’ is intimately connected with his understanding of the nature of human beings. Aristotle may have never actually said in so many words that human beings are best defined as rational animals, but there can be little doubt that were he asked he would have accepted the definition. Be that as it may, many ancient philosophers accepted the claim as authentic Aristotelianism, and it is one which Maimonides certainly adopted.^20^ To be rational for the medievals is not only to exercise rational thought, but to *know* the truths arrived at rationally. Maimonides’ adoption of this position had momentous implications for his thought, leading to many of his more unusual positions.

These include:

- universalism: maximizing one’s natural intellectual abilities; neither birth nor behavior is what makes one a fully-fledged human being.
- an instrumental view of the commandments of the Torah: they were tools given to perfect humans morally and socially, a prerequisite for achieving the truly human end of rational understanding, not as ends in themselves, or as theurgically effective instruments (and since they are tools, there is no reward, in the commonly accepted sense of the term, for their fulfillment, and no punishment, in the commonly accepted sense of the term, for their violation).
- elitism: most (potential) human beings, because of laziness or force of circumstances, remain unfulfilled in their humanity and ought to serve as instruments in the hands of those who have reached philosophical enlightenment (who in turn ought to seek to imitate God and guide and protect the intellectually less fortunate).
- esotericism: for their own good, the masses must be protected from exposure to the truths understood after much effort by the intellectually more perfected.

None of these positions had much impact on Judaism after Maimonides, and many people today who revere his memory and devote themselves to the study of his *Mishneh Torah* would probably deny that he held them. But one consequence of his acceptance of Aristotle’s definition of human beings had a dramatic impact on subsequent Jewish self-understanding: the unprecedented idea that Judaism has dogmas in the strictest sense of the word. Since humans are defined as such by what they know, the Torah must teach truths. From here it is a short step to systematic theology (absent from rabbinic writings) and from systematic theology it is a short step to dogmatics (equally absent from rabbinic writings).

Maimonides opened his magisterial law code, *Mishneh Torah* with the following statement (here translated loosely):

The most important principle of all the principles of the Torah, and the fundamental axiom of all the sciences, is the same, to wit, to know that there exists a First Existent, that It gives existence to all that exists, and that all existent beings, from the heaven to the earth and what is between them, exist only due to the truth of Its existence.

Knowing this, Maimonides goes on to say, is a positive commandment – indeed the first positive commandment in his *Book of Commandments*, not to mention the first of the “Thirteen Principles”.

In making these claims Maimonides imports science (in the guise of *ma’aseh bereshit*, Greek physics, and *ma’aseh merkavah*, Greek metaphysics) into the very heart of Torah. Indeed the twentieth century’s leading Maimonidean, Rabbi Josef Kafih, went so far as to deny the possibility of secular studies (*limmudei hol*) for Maimonides: if a discipline yields truth, it is not secular.^21^

Moreover, to know something, for Maimonides (following Aristotle), is to know it through or with its causes. The first commandment of the Torah is to *know* that God exists; and, as Maimonides makes clear in the Introduction to the *Guide of the Perplexed*, the only way to fulfill that commandment is through the study of physics and metaphysics.

The implications of this are vast:

- The study of science becomes incumbent upon all Jews who want to fulfill even the first commandment of the Torah.
- Psychoanalysis may be a Jewish science, as its opponents claimed, and Lysenko’s biology was certainly socialist “science”, but surely no reader of this journal would claim that there can be a *Jewish* physics or *Jewish* metaphysics. Thus, the science which Jews are commanded to study is precisely that science which is taught (for Maimonides) by uncircumcised Greeks and oppressive Muslims.
- One who has mastered what Maimonides calls (in the Introduction to the *Guide of the Perplexed*) the legal science of the Torah (i.e. the Talmudist) is thus inferior to one who has mastered the secrets of the Torah, i.e. the person who understands physics and metaphysics. (It is no wonder that many who read Maimonides expostulate: “This is Greek to me!” and that medieval rabbis wanted to burn or at least excise the 51st chapter of the third part of the *Guide*.^22^)
- Truth is absolute and objective; there can thus be no such things as intellectual (or spiritual) authority per se. Statements are true irrespective of the standing of the person making them. Maimonides could thus have no patience for the sorts of claims to rabbinic authority which underlie the contemporary doctrine of *da’at Torah* (charismatic rabbinic authority) in its various permutations.

One might expect that belief in one God Who created all human beings in the divine image should lead to a universalist ethic, according to which all human beings are – in principle – equal in the eyes of God and equally beloved by God. Maimonides, unlike many Jews, Christians, and Muslims over the last two millennia, actually accepts the universalist implications of the belief that humans are created in the image of God. But he couples that with acceptance of a hard-edged philosophical elitism. In his eyes, creation in the image of God is a challenge, not an endowment. Those who fail to rise to the challenge allow their potential for God-likeness to go to waste, and die as they were born, as only potentially human. Those who meet the challenge may be called the elect and are, in effect, “God-liked” (to use an expression Maimonides would himself never have used!).

In the history of Judaism very few figures were as consistently and emphatically universalist as Maimonides. The Torah is true, he held, and is certainly the most effective route to human perfection, but it is not the only route. It is the most effective route for the following reason. One cannot achieve perfection as a human being (i.e. deep understanding of the world created by God, and hence of God, to the extent that such understanding is possible) without first achieving a very high level of moral perfection. God, as our Creator, knows us best and knows what is best for us, and thus God’s Torah is certainly the best way to achieve that perfection. But it is not the only way. An enthusiastic Maimonidean such as Jacob Anatoli (thirteenth century) understood the implications of this clearly: in his eyes a scientifically trained Gentile is superior to a punctilious Jew who has no scientific training.

Maimonides was a rationalist, a universalist, an elitist, but also, in his own eyes, a proud Jew.

## Supplementary Material



## References

[1] Kellner M (1986). Dogma in Medieval Jewish Thought.

[2] Kellner M (2006). Must a Jew Believe Anything?.

[3] Berger D (2008). The Rebbe, the Messiah, and the Scandal of Orthodox Indifference.

[4] Kellner M (2006). Maimonides’ Confrontation with Mysticism.

[5] Idel M, Idel M (1990). From Jewish Esotericism to European Philosophy: An Intellectual Profile of Kabbalah as a Cultural factor. Kabbalah: New Perspectives.

[6] Berger D (1991). On Some Ironic Consequences of Maimonides’ Rationalist Messianism. Maimonidean Studies 2.

[7] Kellner M, Saperstein M (1992). Messianic Postures in Israel Today. Essential Papers on Messianic Movements in Jewish History.

[8] Kellner M (2010). Science in the Bet Midrash: Studies in Maimonides.

[9] Lasker DJ (1990). Proselyte Judaism, Christianity, and Islam in the Thought of Judah Halevi. Jewish Quarterly Review.

[10] Batnitzky L (2006). Leo Strauss and Emmanuel Levinas: Philosophy and the Politics of Revelation.

[11] Smith S (2009). The Cambridge Companion to Leo Strauss.

[12] (1963). The Guide of the Perplexed by Moses Maimonides, translated by Shlomo Pines.

[13] Nuland SB (2005). Maimonides.

[14] Davidson HA (2005). Maimonides: The Man and his Works.

[15] Kraemer J (2009). Maimonides: The Life and World of one of Civilization’s Greatest Minds.

[16] Maimonides M Commentary to Mishnah Hagigah.

[17] Maimonides M Laws of the Foundations of the Torah.

[18] Maimonides M Introduction to the Guide of the Perplexed.

[19] Maimonides M Laws of the Foundations of the Torah.

[20] Kellner M (2010). Science in the Bet Midrash: Studies in Maimonides.

[21] Kafih J (1987). Secular Studies in the Rambam. Cross-roads: Halacha and the Modern World.

[22] Shem Tov Falaquera (1225–1290). Commentary on the Guide of the Perplexed (Moreh Hamoreh). III:51 (beginning)

