# Supplementary material for: From Moses to Moses
Source: Rambam Maimonides Med J. 2010 Oct 31;1(2):e0013. doi: 10.5041/RMMJ.10013 (PMC3678785; doi:10.5041/RMMJ.10013)
Supplement: Supplementary file 1 [file rmmj-1-2_e0013_supp.pdf]

Supplementary Material

This appendix has been provided by the authors to give readers additional background reading and information about their work.

Supplement to:

Kellner M. From Moses to Moses. RMMJ 2010;1(2):e0013. DOI:10.5041/RMMJ.10013

The interpretation of Maimonides presented here (and since he wrote esoterically, every presentation of his thought is an interpretation) is supported in the following books of mine:

1. *Dogma in Medieval Jewish Thought*. Oxford: Oxford University Press; 1986. (paperback reprint: 2004) (http://www.littman.co.uk/cat/kellner.html)
   Maimonides on Human Perfection. Atlanta: Scholars Press (Brown Judaic Studies); 1990.
2. *Maimonides on Judaism and the Jewish People.* Albany: SUNY Press; 1991.
   Maimonides on the Decline of the Generations and the Nature of Rabbinic Authority. Albany: SUNY Press; 1996.
   Must a Jew Believe Anything? London: Littman Library of Jewish Civilization; 1999. Second edition, revised and expanded: 2006.
3. *Maimonides' Confrontation With Mysticism*. London: Littman Library of Jewish Civilization; 2006.
4. *Science in the Bet Midrash: Studies in Maimonides*. Brighton, MA: Academic Studies Press; 2009.

Further References:

- On the revolutionary character of the *Mishneh Torah*, see Isadore Twersky, *Introduction to the Code of Maimonides (Mishneh Torah)*. New Haven: Yale University Press; 1980.
- For Berger's critique of Habad, see David Berger, *The Rebbe, the Messiah, and the Scandal of Orthodox Indifference*. London: Littman Library of Jewish Civilization; 2001.
- For Idel's understanding of the role of Maimonides in the establishment of Kabbalah in the Jewish world, see Moshe Idel, "Maimonides and Kabbalah". In: *Studies in Maimonides*, edited by Isadore Twersky. Cambridge: Harvard University Press; 1990:31–81.
- For a lively biography of Maimonides, which includes a critique of Leo Strauss's views, see Herbert A. Davidson, *Moses Maimonides: The Man and His Works*. Oxford: Oxford University Press; 2005.
